# Tendon Harvest for Treatment of Radial Nerve Palsy Using Endoscopic Technique

**DOI:** 10.29252/wjps.7.3.332

**Published:** 2018-09

**Authors:** Mohammad-Reza Akhoondinasab, Sharyar Sadeghi, Iraj Mirzaii-Dizgah

**Affiliations:** 1 Department of Plastic and Reconstructive Surgery, Fatimah Zahra Hospital, Iran University of Medical Sciences, Tehran, Iran;; 2 Department of Physiology, School of Medicine, AJA University of Medical Sciences, Tehran, Iran

**Keywords:** Endoscopic technique, Tendon harvest, Radial nerve palsy

## Abstract

**BACKGROUND:**

Endoscopic method for many surgeries with minimal access is proposed to be effective for preventing the excessive scar formation, reducing pain, cosmesis, and the early return to work. Surgical outcomes of endoscopic and open methods for tendon harvest in treatment of radial nerve palsy were evaluated.

**METHODS:**

In a randomized single-blind clinical trial study, 10 patients with radial nerve palsy who referred to the Plastic Surgery Department of the Fatimah Zahra Hospital, Iran University of Medical Sciences, Tehran, Iran were divided into two equal groups. Flexor carpi radialis (FCR), flexor carpi ulnaris (FCU) and the palmaris longus (PL) tendons were harvested by endoscopic or open techniques. The outcomes (tendon harvest time, rate of post-surgical pain, amount of pain medication, patient satisfaction, amount of scar, and length of scar) are measured.

**RESULTS:**

There was no significant difference in time of surgery between two methods. Vancouver scar scale, cosmetic satisfaction, severity of postoperative pain and also drugs for pain relief after tendon harvest surgery were significantly lower in endoscopic method than open method.

**CONCLUSION:**

Regarding the low invasiveness, high satisfaction rate of patients, low pain severity, low scar and the little need for opiate to reduce pain in the endoscopic method, endoscopic tendon harvest technique for radial nerve palsy seems to have priority over open method.

## INTRODUCTION

Upper limb lesions are very common, with approximately 1.3% of body lesions occurring in this limb. Lesions in peripheral nerves including median, ulnar and radial nerves are very common in the upper extremity. So many patients with hand lesions refer to hospitals and rehabilitation centers while among the peripheral nerves, radial nerve is more susceptible to injury.^1^ Radial nerve palsy leads to impotence in the movement of the hand. The patient loses the ability to extend the wrist, fingers and thumb, movements that are essential for functional grasp. In addition, the patient loses grip strength because he/she cannot maintain the wrist during power grip. Fortunately, the loss of cutaneous sensibility in the radial nerve distribution is well tolerated.^2^

Tendon transfers for radial nerve injuries are performed, whether spontaneous or after nerve repair, are no longer expected. Prerequisites for successful transfers should be met, especially tissue equilibrium.^3^ The use of flexor carpiulnaris was developed to restore the extension of metacarpophalangeal joint, though there are barriers to the using flexor carpi ulnaris.^4^ In tendon transfer surgery, which is performed to restore the missing function and performance, a tendon is transferred to the junction of a muscle-tendon unit. Most researchers found that tendon transfer in patients with radial nerve palsy may produce good results once nerve repair has failed. When a recovery in radial nerve lesions is not seen in a one-year period, tendon transfer is recommended.^5^ Most surgeons have used the pronator teres (PT) to extensor carpi radialis brevis (ERCB) transfer to restore wrist extension.^3^

Finger extension restoration may be done using the flexor carpi radialis (FCR), flexor carpi ulnaris (FCU), or flexor digitorum superficialis (FDS).^6^ Traditionally, a long longitudinal incision with extensive dissection has to be performed to harvest the palmaris longus (PL), FCR or FCU tendons and to transfer their to the extensor regions.^7^ This traditional open tendon harvest has a few associated incidences of hypertrophic scarring, scar tenderness, pain, and delayed return to work.^8^ Endoscopic technique for many surgeries with minimal access is proposed to be effective for preventing the excessive scar formation, reducing pain, cosmesis, and the early return to work.^8^^-^^10^ In the current study, we compared surgical outcomes, including time of tendon harvest, objective scar assessment, subjective cosmetic satisfaction, severity of postsurgical pain, and analgesic drugs for pain relief, between endoscopic and open tendon harvest for treatment of radial nerve palsy.

## MATERIALS AND METHODS

This is a randomized single-blind clinical trial study (Clinical Trials.gov ID: IRCT2016102430462N1) including 10 individuals (1 woman and 9 men) aged 15-60 years who had radial nerve palsy without response to primary nerve repair. Patients suffering from radial nerve palsy referred to the plastic surgery department of the Fatimah Zahra Hospital, Iran University of Medical Sciences, Tehran, Iran were invited to participate in the study after full explanation of the aims of the trial in local dialect. The formal recruitment process was completed when patients became fully aware of the study consent form and signed it.

Inclusion criteria was patients with an age range 15-60 years with traumatic radial nerve injury and exclusion criteria were patients with non-traumatic radial nerve injury, patients who have other peripheral nerve injury and patients treated with corticosteroids. In this study, the outcomes such as tendon harvest time, rate of post-surgical pain, amount of pain medication, patient satisfaction, amount of scar, and length of scar, are measured. All procedures are performed by a single surgeon. At the end of surgery the person responsible for data collection related to study without having knowledge of the patient and surgical procedure, collect information. 

In the endoscopic surgery method, two cm transverse incision was done in the volar site of wrist. Then endoscopic port was inserted through incision and under endoscopic magnification, FCU (or FCR) and PL tendons were harvested. In the open surgery method, 9-12 cm longitudinal incision was done in the volar site of forearm according to a classic method. Other processes of surgery were the same in both methods. In both methods the FCU (or FCR if patients had a posterior interosseous nerve palsy) and PL were readily accessible beneath the full-thickness volar forearm flap. 

Branches of the radial sensory nerve and the radial artery are identified and protected. Tendon transfers of the thumb and finger extensors are performed before transfers about the wrist. This sequence allows the surgeon to judge sufficient tension using wrist motion. The extensor pollicis longus (EPL) tendon is woven into the PL and the FCR or FCU are woven into the extensor digitorum communis (EDC) using a tendon braider. Tension is adjusted until wrist flexion of 30° produces adequate thumb and finger extension via tenodesis and wrist extension allows passive finger flexion into the palm. Once digital extension transfers are completed, the PT is woven into the ECRB. Tension is adjusted until a 30° extension resting posture of the wrist is achieved. All Pulvertaft tendon passes are secured with braided non-absorbable suture. Once skin flaps are closed, the extremity is splinted with the wrist in 30° of extension, the metacarpophalangeal (MCP) joints in full extension, and the thumb abducted with the interphalangeal (IP) joint in extension.

A scar was analyzed objectively using the Vancouver Scar Scale (VSS) at 6 months postoperatively. VSS is a validated objective scar scale that is commonly used in clinical studies. VSS assesses four domains: vascularity, pigmentation, pliability, and height of the scar. Vascularity, pigmentation, and height of the scar are scored from 0 to 3, and pliability is scored from 0 to 5. The score is the sum of four domains, with a score of 0 representing normal skin and a score of 14 representing the worst scar.^11^

Cosmetic satisfaction was assessed with four questions: (1) Do you think others look at your forearm scar? (2) Do you try to conceal your scar? (3) Does your forearm scar influence your choice of clothes? (4) How often do you think of your scar? Each question was scored as 0 (never), 1 (sometimes), 2 (frequently), or 3 (always). Total cosmetic satisfaction score was defined as the sum of scores (four questions).^12^

The pain intensity produced by hypertonic saline injections was assessed on a 10 cm electronic visual analogue scale (VAS) which could be adjusted by using an external handheld slider. The VAS was anchored with ‘no pain’ and ‘maximum pain’, 0 cm and 10 cm, respectively. Analyses were performed using SPSS software version 18 (SPSS Inc. Chicago, IL, USA). The statistical analysis of t-test, Mann-Whitney test and χ^2^ were used. P < 0.05 was considered statistically significant.

## RESULTS

Demographics characteristics are listed in Table 1. Age, gender and nerve injury level of the patients were not significantly different between two methods (Table 1). Student t-test showed that there was no significant difference in time of surgery between two methods (*p*=0.127; Table 2). Mann-Whitney test showed that Vancouver scar scale (VSS) and cosmetic satisfaction of patients were significantly lower in endoscopic method than open method (*p*=0.008 and 0.007 respectively; Table 2). χ^2 ^test indicated that severity of postoperative pain and also drugs for pain relief after tendon harvest surgery were significantly low in endoscopic method than open method (*p*=0.007 and 0.036 respectively; Table 2). 

**Table 1:** Demographic characteristic of patient in open and endoscopic surgery methods of tendon harvest

**Variable**	**Open method**	**Endoscopic method**	**P value**
Age (year) (mean±SD)	36.2±13.0	28.2± 6.1	0.248
Sex (Male/female)	4.1	5.0	0.5
Level of nerve injury (Arm/Forearm )	3.2	4.4	0.5

**Table 2:** The outcome of endoscopic versus open tendons harvest for the treatment of radial nerve palsy

**Variable**		**Open method**	**Endoscopic method**	**P value**
Tendon harvest time (min) (mean±SEM)		15.0±3.2	28.0±6.9	0.127
Vancouver scar scale [median (IQR)]		5 (2.5)	1 (1)	0.008 *
Cosmetic satisfaction [median (IQR)]		8 (4)	0 (0)	0.007*
Severity of postoperative pain (%)	Mild	0%	60%	0.007*
	Moderate	0%	40%	
	Severe	100%	0%	
Drugs for pain relief (%)	Novafen	0%	20%	0.036*
	Novafen+Apotel	0%	60%	
	Novafen+Apotel+Tramadol	100%	20%	

IQR=Inter quartile range

## DISCUSSION

Endoscopic technique for surgeries has the benefit over open method of reduced tissue trauma and postoperative morbidity.^13^^,^^14^ The purpose of this study was to compare the outcome of endoscopic versus open tendons harvest for the treatment of radial nerve palsy. Patients with this condition were randomized to undergo either endoscopic or open tendon harvest. The principal findings of this study were that endoscopic method of FCU/FCR and PL tendons harvest provided a faster recovery of pain, less drugs for pain relief after tendon harvest surgery, more patient satisfaction, and low VSS after 6 months of tendon harvest surgery to the operated patients as compared with open technique. To best of our knowledge, there was no study about endoscopic FCU/FCR and PL tendons harvest surgery for treatment of radial nerve palsy and this is the first. 

Open method of FCU/FCR and PL tendons harvest method had a higher pain score as compare to endoscopic method in this study. The increased pain could be explained by the extended surgical trauma that the open technique provides compared with the endoscopic technique. Moreover, during the open technique, subcutaneous tissue of the forearm is also incised. That is not the case in endoscopic release because the forearm is incised from its distal aspect and the most forearm tissues remain intact. Related literature has confirmed the extended injury after open release.^15^^-^^17^

In this study, cosmetic satisfaction score was significantly low in endoscopic method than open method after 6 months of tendon harvest surgery. It showed that the satisfaction of patients was higher in the endoscopic method than in the open method. This finding was in accordance with result of other minimally invasive surgery as compare to traditional method were done in other region of body.^17^^-^^19^ The reasons to good satisfaction in the endoscopic method compare to the open method may be good wound cosmetics in patients, life quality improvement, less pain, and early return to work due to minimally invasive procedure in endoscopic method. 

Over half of patients who undergo common, outpatient, elective hand surgery procedures receive prescriptions for opioid analgesics. It has been shown that the need for opioid analgesics is closely associated with the type of procedure that is performed. It has also been demonstrated that the prevalence of opioid use and dependence among patients undergoing surgery procedures is rising. Deaths related to opioid prescriptions have increased over recent years.^20^ Therefore, it is recommended that the use of opioids should be limited for pain relief after surgery. As there is no need to use opioids in patients with endoscopic method, it seems that this method is suitable than open method for treatment of radial nerve palsy. 

VSS assesses 4 variables: vascularity, height/thickness, pliability, and pigmentation and a score of 0 representing normal skin and a score of 14 representing the worst scar.^11^ Total scores for VSS was significantly lower in endoscopic than open method in this study. It shows that the endoscopic method is proposed to be effective for preventing the excessive scar formation. Regarding the low invasiveness, high satisfaction rate of patients, low pain severity, low scar and the little need for opiate to reduce pain in the endoscopic method, endoscopic tendon harvest technique for radial nerve palsy seems to have priority over open method.

## CONFLICT OF INTEREST

The authors declare no conflict of interest.
